# Acute Ischemic Stroke as the Initial Presentation of Primary Sjögren’s Syndrome: A Case Report

**DOI:** 10.7759/cureus.106755

**Published:** 2026-04-09

**Authors:** Yuvrajsing K Pakal, Shreya N Gade, Neha P Dharap

**Affiliations:** 1 Department of Medicine, Smt. Kashibai Navale Medical College and General Hospital, Pune, IND

**Keywords:** autoimmune vasculitis, ischemic cerebrovascular disease, ro-52 antibodies, sjögren’s syndrome, young onset stroke

## Abstract

Sjögren's syndrome is a long-standing autoimmune condition that predominantly affects the body's exocrine glands, most notably the eyes and mouth, leading to ocular and oral dryness. Neurological involvement, particularly affecting the central nervous system, is infrequently encountered, and the occurrence of stroke as an initial clinical manifestation remains exceptionally uncommon. We report a 42-year-old diabetic male who presented with an acute onset of right hemiplegia and aphasia, and was subsequently diagnosed with primary Sjögren's syndrome. Evaluation revealed multiple acute infarcts in the left middle cerebral artery territory with apical clot and ischemic heart disease. Positive antinuclear antibody by immunofluorescence, Ro-52 recombinant antibody positivity, and raised inflammatory markers suggested an autoimmune vasculitic process secondary to Sjögren's syndrome. The patient improved significantly after pulse methylprednisolone and hydroxychloroquine therapy. This case highlights the importance of considering autoimmune etiologies like Sjögren's syndrome in young stroke patients with systemic features.

## Introduction

Sjögren's syndrome is a chronic autoimmune disorder characterized by lymphocytic infiltration of salivary and lacrimal glands, leading to its classic manifestations of persistent oral dryness (xerostomia) and ocular dryness (keratoconjunctivitis sicca). The disorder shows a marked female predominance, affecting women far more often than men, with a ratio close to 9:1. Although glandular dysfunction forms the clinical foundation of the disease, Sjögren's syndrome is increasingly recognized as a systemic illness, capable of involving multiple organ systems [1].

Neurological complications, while established in the disease spectrum, occur in a relatively small subset of patients. Peripheral nervous system involvement represents the majority of these cases (approximately 65%), whereas central nervous system manifestations are far less frequent (around 12.5%) [2] and clinically heterogeneous, including cognitive impairment, transverse myelitis, aseptic meningitis, and demyelinating syndromes [3]. Cerebrovascular events, particularly ischemic stroke as an initial presentation, are exceptionally uncommon and are thought to result from immune-mediated vascular injury, including small- and medium-vessel inflammation, endothelial dysfunction, and immune complex deposition.

Such presentations may obscure recognition of the underlying autoimmune process, particularly in younger individuals lacking conventional vascular risk factors [4]. Identifying autoimmune causes in atypical stroke presentations is crucial, as timely immunosuppressive therapy can substantially modify disease trajectory and improve neurological outcomes [5].

Here, we report a case of a 42-year-old male with type 2 diabetes mellitus who presented with acute ischemic stroke and was subsequently diagnosed with probable primary Sjögren's syndrome based on clinical features and serological findings. Young-onset stroke (age <45 years) warrants evaluation for uncommon etiologies, including autoimmune disorders and vasculitic processes, particularly in the absence of typical risk factors.

## Case presentation

A 42-year-old male, a known case of type 2 diabetes mellitus on oral hypoglycemic agents, presented with sudden-onset aphasia and right-sided weakness for the past nine hours, associated with altered sensorium. There was no history of convulsions, headache, vomiting, or trauma.

Relatives reported recent dryness of the eyes and mouth before the onset of neurological symptoms. An annular erythematous rash was noted over the trunk and body. There was no history of photosensitivity, joint pain, or oral ulcers.

On examination, the patient was drowsy, aphasic, and not obeying commands. Vital signs were as follows: body temperature of 37.1°C; pulse rate of 90 beats per minute, regular; blood pressure of 160/90 mmHg; and respiratory rate of 16 breaths per minute. Neurological examination noted the following findings: right-sided hemiplegia (power 0/5), right upper motor neuron facial palsy, brisk deep tendon reflexes on the right, and an extensor plantar response on the right. The Schirmer test was positive, confirming reduced tear production. Skin showed annular erythema over the trunk and limbs. Oral mucosa and conjunctival dryness were present. Other systemic examinations were unremarkable.

A clinical diagnosis of large acute ischemic stroke was made. The patient was started on intravenous mannitol 100 mL three times daily, aspirin 75 mg once daily, and enoxaparin 0.4 mL once daily. These were initiated for secondary stroke prevention in a setting of suspected cardioembolic stroke due to the presence of a left ventricular apical thrombus on echocardiography.

Routine investigations, including hemogram, liver function tests, renal function tests, and serum electrolytes, were within normal limits. Other investigations, such as C-reactive protein (CRP), glycated hemoglobin (HbA1c), and erythrocyte sedimentation rate (ESR), were also conducted (Table 1).

**Table 1:** Summary of laboratory investigations and their results. Reference values were obtained from standard laboratory reference ranges [6].

Parameter	Result	Reference range
Erythrocyte sedimentation rate (ESR)	15 mm/hr	<15 mm/hr
C-reactive protein (CRP)	48 mg/L	<10 mg/L
Glycated hemoglobin (HbA1c)	9%	4-5.9%

**Figure 1:**
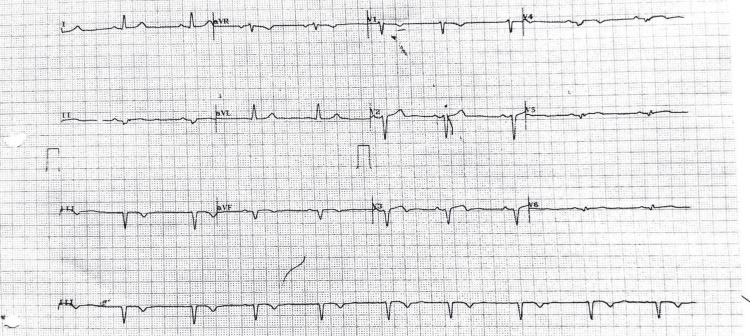
Twelve-lead ECG showing Q waves in inferior leads and poor R-wave progression in precordial leads.

Abdominal ultrasonography (USG) showed splenomegaly. Electrocardiogram (ECG) showed Q waves in inferior leads (II, III, aVF) and poor R-wave progression in precordial leads, suggestive of prior inferior wall myocardial infarction (Figure 1).

2D echocardiography revealed a left ventricular apical clot (16 × 16 mm) with distal septo-apical and apico-lateral akinesia and grade I diastolic dysfunction. The left ventricular ejection fraction (LVEF) was reduced to 35%, consistent with ischemic cardiomyopathy. Left heart catheterization was not performed due to clinical considerations and the presence of a clearly identified left ventricular thrombus.

**Figure 2:**
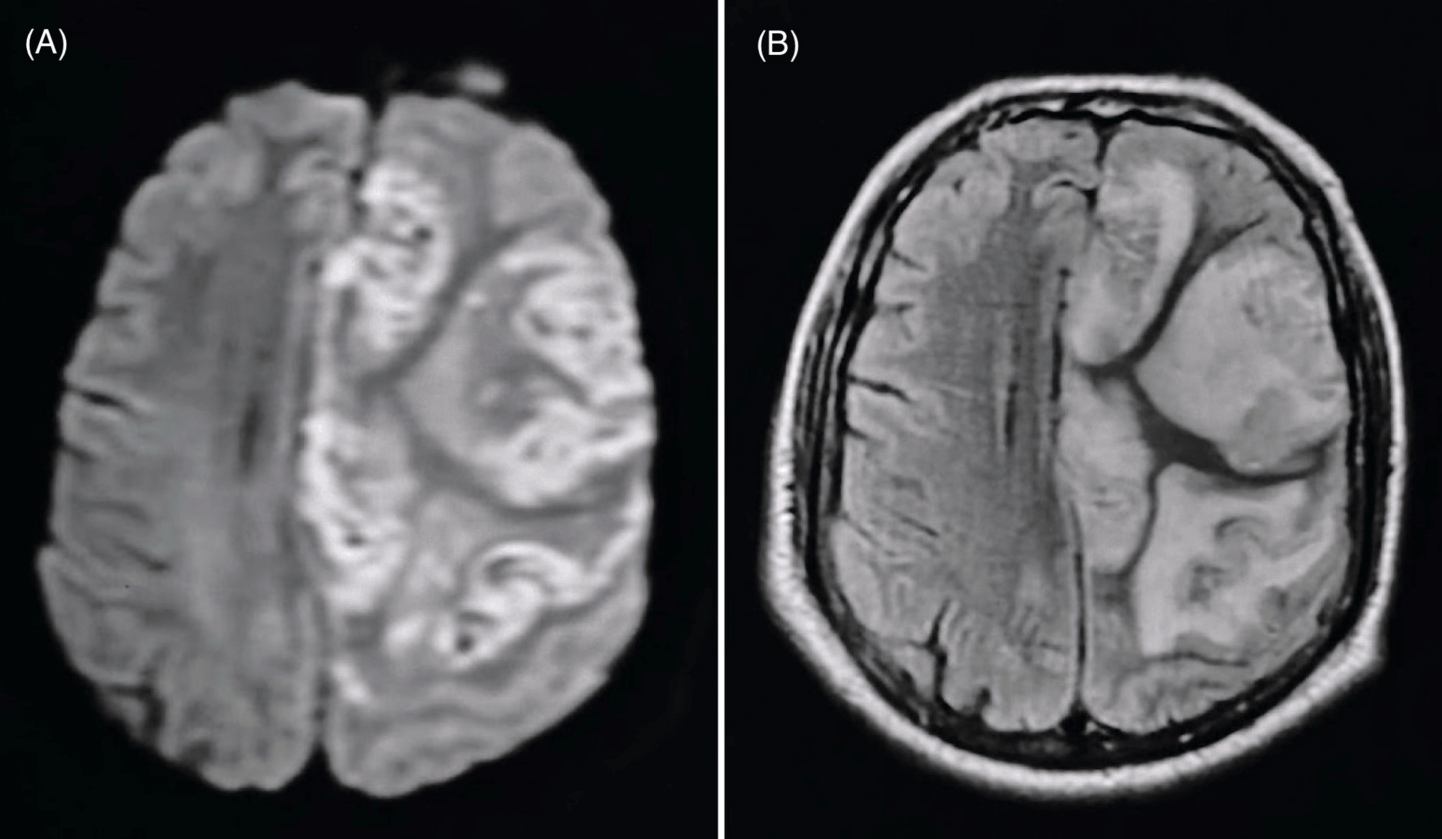
MRI of the brain showing hyperintense lesions in the left middle cerebral artery territory suggestive of acute infarcts. (A) Diffusion-weighted imaging (DWI) sequence. (B) Fluid-attenuated inversion recovery (FLAIR) sequence.

Magnetic resonance imaging (MRI) of the brain demonstrated large acute infarcts in the left fronto-parietal cortex, corpus callosum, perirolandic region, and right cingulate gyrus, corresponding to the middle cerebral artery (MCA) territory. Chronic small-vessel ischemic changes were also noted (Figure 2).

Given the patient’s relatively young age and the presence of multi-territorial infarcts on neuroimaging, an evaluation for autoimmune and prothrombotic causes was undertaken. Autoimmune serological investigations, including rheumatoid factor, antinuclear antibody (ANA) by immunofluorescence, ANA blot, and antiphospholipid antibody, were conducted (Table 2).

**Table 2:** Summary of autoimmune serological investigations. SSA: Sjögren's-syndrome-related antigen A; SSB: Sjögren's-syndrome-related antigen B.

Parameter	Result
Antinuclear antibody (ANA) by immunofluorescence	Positive
ANA blot	Ro-52 recombinant antibody positive; SSA and SSB negative
Antiphospholipid antibody (APA) IgG	Negative
Rheumatoid factor	Positive

In view of positive autoimmune markers, serum homocysteine levels were evaluated and were found to be elevated at 19.8 µmol/L (reference range: 4-14 µmol/L) [6].

**Figure 3:**
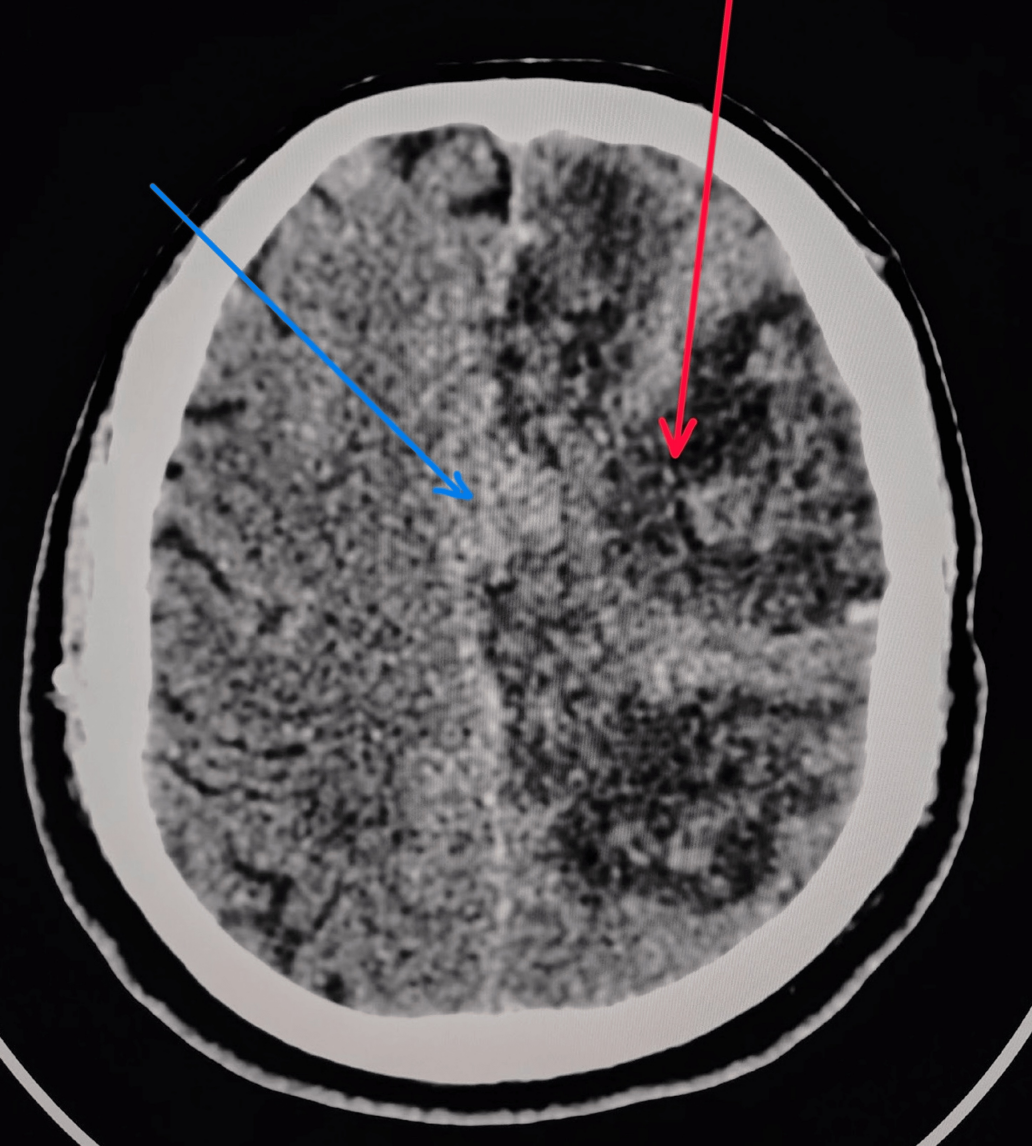
Repeat non-contrast CT of the brain showing hemorrhagic transformation (red arrow) of the infarct with a midline shift (blue arrow) of 7.5 mm, following clinical deterioration after 48 hours.

After 48 hours, the patient's sensorium deteriorated. A repeat non-contrast CT of the brain (Figure 3) revealed hemorrhagic transformation with a midline shift of 7.5 mm, following which enoxaparin was withheld. The patient's condition improved after initiating intravenous methylprednisolone therapy.

Considering the young age, multi-territorial infarcts, positive ANA with Ro-52 antibody positivity, and features of xerostomia and xerophthalmia, a diagnosis of probable primary Sjögren’s syndrome presenting as autoimmune-mediated ischemic stroke was made.

The patient was treated with pulse intravenous methylprednisolone (1 g daily for five days) and aspirin 75 mg once daily, followed by oral prednisolone 40 mg once daily and hydroxychloroquine 200 mg once daily. Rivaroxaban was initiated for the management of the left ventricular thrombus.

Over the following two weeks, the patient showed complete improvement in consciousness and marked improvement in motor function. Speech recovered gradually, with full normalization after two weeks, and the right-sided motor power improved to 3/5. The patient was discharged on oral steroids, hydroxychloroquine, antiplatelets, statins, oral hypoglycemics, and anticoagulation. The patient has been on regular follow-up for the past two months with sustained clinical improvement.

## Discussion

Sjögren’s syndrome is a chronic autoimmune disorder primarily characterized by lymphocytic infiltration of exocrine glands, resulting in xerostomia and keratoconjunctivitis sicca. However, it is increasingly recognized as a systemic disease with a wide range of extraglandular manifestations, including neurological involvement. Central nervous system (CNS) manifestations are relatively uncommon but clinically significant, and ischemic stroke as an initial presentation is rare [1].

In the present case, a 42-year-old male presented with an acute ischemic stroke involving multiple vascular territories. Although type 2 diabetes mellitus and elevated homocysteine levels are established vascular risk factors, they typically result in small vessel or single-territory infarcts [7,8]. The presence of multi-territorial infarcts involving the corpus callosum and contralateral regions suggested a more complex etiology.

A cardioembolic source was strongly supported by the presence of a left ventricular apical thrombus and reduced ejection fraction (35%). While cardioembolic strokes commonly involve large vascular territories, the distribution of infarcts in this case was not entirely typical of a single embolic event, suggesting the possibility of overlapping mechanisms contributing to the patient’s presentation [9].

In addition, the patient demonstrated sicca symptoms, a positive Schirmer test, and immunological markers, including ANA positivity, rheumatoid factor positivity, and anti-Ro52 antibody positivity, supporting the presence of an underlying autoimmune process. However, according to the 2016 American College of Rheumatology (ACR)/European League Against Rheumatism (EULAR) classification criteria for primary Sjögren’s syndrome, greater diagnostic weight is assigned to anti-SSA/Ro antibody positivity and labial salivary gland biopsy findings [10]. In this case, anti-SSA antibodies were negative, and a salivary gland biopsy was not performed, limiting the ability to establish a definitive diagnosis. Therefore, this case is more appropriately described as probable primary Sjögren’s syndrome.

Previous studies have reported that autoimmune-mediated vascular injury, including small- and medium-vessel vasculitis, endothelial dysfunction, and immune complex deposition, may contribute to ischemic events in Sjögren’s syndrome [11]. Anti-Ro52 antibodies have also been associated with increased risk of cerebral small vessel disease and poorer neurological outcomes, suggesting a potential role in vascular injury [12].

Taken together, the presence of multiple contributing factors, including diabetes mellitus, elevated homocysteine levels, left ventricular thrombus, and autoimmune markers, supports a multifactorial etiology, with cardioembolism likely playing a major role and a possible autoimmune contribution.

This case is notable for several reasons. First, stroke as an initial presentation of Sjögren’s syndrome remains rare, with only a limited number of cases reported and very few involving major cerebral vessels. For instance, a recently reported case described a young female patient presenting with right middle cerebral artery infarction as the first manifestation of Sjögren’s syndrome [13]. In contrast, our case involves a male patient with multi-territorial infarcts at initial presentation, highlighting a broader and less typical pattern of neurological involvement. Additionally, this case underscores the importance of considering autoimmune etiologies in young stroke patients with atypical imaging or systemic features.

## Conclusions

This case highlights a rare presentation of acute ischemic stroke in a relatively young patient with features suggestive of Sjögren’s syndrome. While a cardioembolic source was strongly supported by the presence of a left ventricular thrombus and reduced ejection fraction, additional factors such as diabetes and elevated homocysteine likely contributed.

Sjögren’s syndrome may therefore represent a possible contributing factor rather than a definitive cause, supporting a multifactorial etiology. Clinicians should consider autoimmune causes in young or atypical stroke presentations.
